# Mit-O-My I Can't Breath! Mitomycin C–Induced Pneumonitis Leading to Acute Respiratory Distress Syndrome, a Rare Case

**DOI:** 10.1155/crom/5587748

**Published:** 2025-06-23

**Authors:** Tyler Steve, Prarthna V. Bhardwaj

**Affiliations:** ^1^Department of Internal Medicine, University of Massachusetts Chan Medical School, Baystate Medical Center, Springfield, Massachusetts, USA; ^2^Department of Hematology Oncology, Baystate Medical Center, Springfield, Massachusetts, USA

## Abstract

Mitomycin C (MMC) pneumonitis leading to acute respiratory distress syndrome (ARDS) is a rare and life-threatening adverse reaction to MMC. Diagnosing MMC pneumonitis can be challenging as more frequent etiologies such as bacterial infections are often targeted first due to patients being immunocompromised from chemotherapy. We report a case of a middle-aged male who was administered MMC without concomitant vinca alkaloid, who developed ARDS secondary to MMC pneumonitis requiring intubation and intensive care. The patient recovered with steroid treatment after being on antibiotics for many days, and no infectious etiology was ever identified. This case emphasizes the importance of recognizing MMC as a potential cause for pneumonitis which can lead to ARDS and death.

## 1. Introduction

Mitomycin C (MMC) is an alkylating agent that produces crosslinking in DNA between mostly the guanine and cytosine pairs, which leads to disruption of DNA and RNA synthesis. Pulmonary toxicity is associated with MMC; however, the frequency is thought to be between 2% and 12% [1]. Studies suggest that MMC lung toxicity is dose dependent, occurring at cumulative dose levels greater than 20 mg/m^2^ [1]. Additionally, the majority of reported cases of lung toxicity secondary to MMC are also with concomitant use of vinca alkaloids, which suggests a possible synergistic effect [2, 3]. We report a rare case of acute respiratory distress syndrome (ARDS) caused by MMC-induced pneumonitis in a patient with no concomitant vinca alkaloid therapy.

## 2. Case

A 54-year-old male presented to the emergency department with dyspnea on exertion and hypoxia. His personal medical history is significant for advanced chronic obstructive lung disease, alcohol use disorder, and high-grade muscle invasive urothelial carcinoma of the bladder, invasive into the lamina propria, but not the muscularis propria. He was treated with concurrent chemoradiation therapy with MMC and 5-fluorouracil. His chemotherapy was a total of one cycle over 49 days, with MMC 12 mg/m^2^, IV push Day 1, and 5-fluorouacil 2500 mg/m^2^, over 120 h on Days 1 and 22. Treatment was started in early August 2016, and the patient now presents to the emergency department 3 months later in November 2016.

Upon presentation to the emergency department, the patient had shortness of breath and cough for the past 3 days with a productive white and yellow sputum. He also complained of intermittent chest pain characterized as a pressure that was worse when coughing. He denied any fever, weakness, neurological changes, or abdominal pain. Vital signs upon arrival were heart rate 93 and blood pressure 107/64, saturating at 99% on nonrebreather with saturation of 80% noted on room air. Physical examination was largely unremarkable, with lungs clear to auscultation, nonlabored respirations, symmetrical chest wall expansion, nondistended and nontender abdomen, regular rate and rhythm, and no focal neurological deficits.

**Figure 1:**
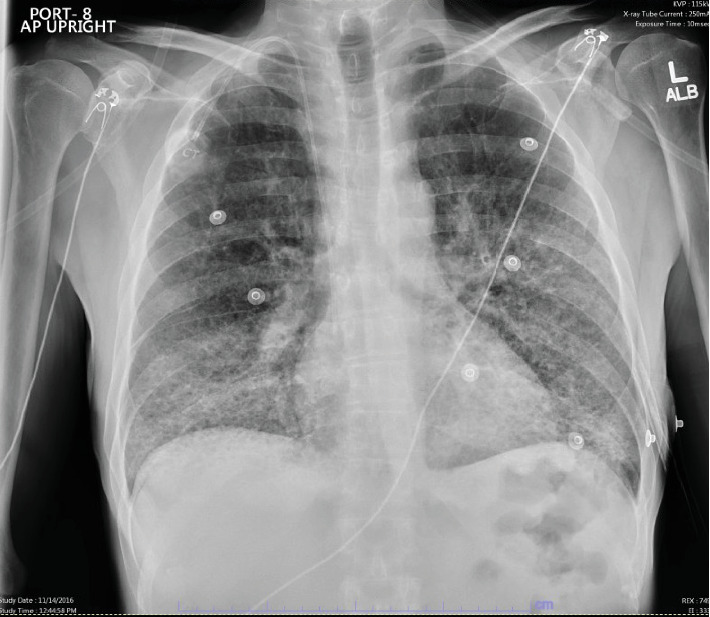
Chest radiograph upon initial presentation to the emergency department. Bilateral interstitial and alveolar airspace disease, left greater than right. Appearance is most suggestive of multilobar pneumonia. This does not have the typical appearance of CHF. Pulmonary hemorrhage or other infectious/inflammatory processes are also on the differential.

**Figure 2:**
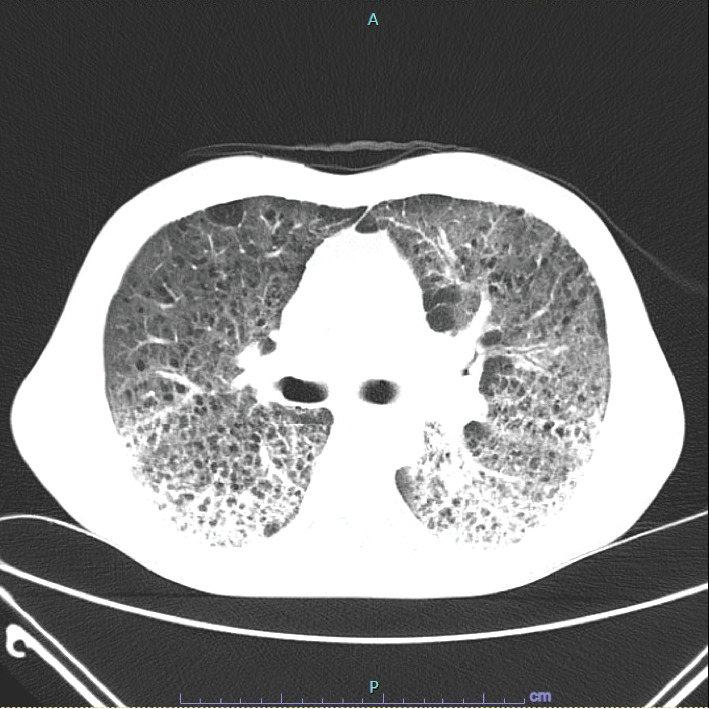
Computed tomography angiogram of the chest. Extensive abnormal appearance of the lung parenchyma, with innumerable small cysts scattered throughout the lung parenchyma and diffuse ground-glass opacities which are slightly more dense and patchy at the lung bases. There is also new adenopathy in the mediastinum and right hilum, likely reactive. Findings are new since August and are of unclear etiology. In a patient who has been recently treated with chemotherapy, this may represent an atypical pulmonary infection such as *Pneumocystis carinii* pneumonia, which has been reported in immunocompromised patients with malignancy. Interstitial pneumonitis related to chemotherapy is also a consideration. A chronic process is not likely given the rapid evolution since August.

He underwent initial lab testing which showed a negative urinalysis, and the complete blood count showed a leukocytosis of 16.6 k/mm^3^, hemoglobin of 10.4 Gm/dL, and hematocrit of 30.8%. An initial chest X-ray showed concern for multilobar pneumonia (Figure 1). The patient underwent computed tomography angiogram (CTA) of the chest which had extensive abnormalities of lung parenchyma, many small cysts, and diffuse ground-glass opacities concerning for atypical pneumonia versus hypersensitivity pneumonitis (Figure 2). The patient was treated with vancomycin, piperacillin/tazobactam, azithromycin, and dexamethasone. Trimethoprim/sulfamethoxazole was added to cover for potential *Pneumocystis* pneumonia (PCP) infection.

He was transferred to the intensive care unit (ICU) on Hospital Day 2 due to worsening hypoxemic respiratory failure, which required intubation. He was also started on vasopressors for hemodynamic support. Bronchoscopy was performed with bronchoalveolar lavage and cultures. Bronchoalveolar lavage fluid reported a hazy pink color with WBC 90 cu mm, RBC 2100 cu mm, and segmented fluid 47%. A viral respiratory panel, strep and legionella antigen testing, *Pneumocystis carinii* staining, HIV testing, and blood cultures all resulted negative. Gram stains of bronchoalveolar lavage fluid from bronchoscopy were negative as well. During his hospitalization, he went on to develop severe ARDS. He was switched to prednisone 1 mg/kg dosing. Given ongoing hypoxemia with no clear diagnosis, he underwent a transbronchial biopsy. Histology from the lung biopsy showed a small fragment of lung parenchyma with reactive changes of alveolar lining cells and minimal chronic inflammation, bronchial mucosa and submucosa with mild chronic inflammation, and no intra-alveolar exudate suggestive of *Pneumocystis*. Cytology studies were negative for malignant cells. With the initiation of prednisone, there was a notable improvement in his overall clinical course. By Day 7 in the ICU, he was extubated. Prednisone was subsequently tapered. Pathology results from the transbronchial biopsy returned negative for PCP. A diagnosis of drug-induced pneumonitis secondary to MMC was made. He was eventually discharged from the hospital on a prolonged prednisone taper and sent to a rehabilitation facility.

**Figure 3:**
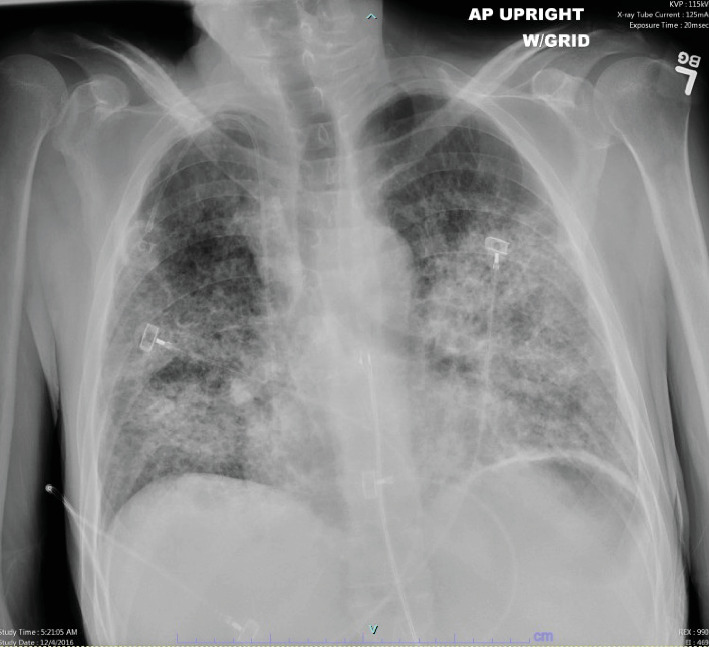
Chest radiograph on readmission to hospital showing stable diffuse interstitial and alveolar opacities predominantly in the mid-lower lungs.

**Figure 4:**
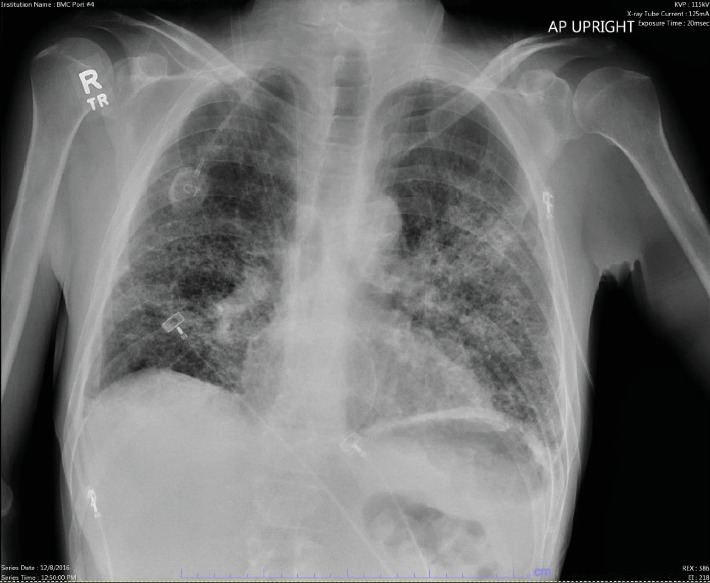
Chest radiograph showing decreased infiltrates after increased doses of steroids.

While still on the prednisone taper at 10 mg daily, he was rehospitalized 7 days after discharge for hypoxia and acute respiratory failure requiring high-flow nasal cannula oxygen supplementation. Initial lab work showed a leukocyte count of 12.6 k/mm^3^, bicarb of 32 mmol/L, lipase of 10 units/L, AST of 24 units/L, ALT of 25 units/L, and Nt-Probnp of 243 pg/mL. He was started on levofloxacin and trimethoprim/sulfamethoxazole as well as methylprednisolone 40 mg every 6 h and albuterol/ipratropium nebulizer as needed. Pulmonology was consulted and had low suspicion for infectious etiology, and patient's steroids were increased. Repeat chest radiograph on this admission showed stable diffuse interstitial alveolar opacities (Figure 3). He continued to have persistent episode of dyspnea, and on Hospital Day 3, methylprednisolone was increased to 60 mg every 6 h. On Hospital Day 5, he was transitioned to Solu-MEDROL 60 mg three times a day and a repeat chest radiograph was obtained showing improvement of interstitial infiltrates (Figure 4). The patient continued to gradually improve, and by Hospital Day 10, he was eventually discharged back to his rehabilitation facility continuing trimethoprim/sulfamethoxazole and a prolonged prednisone taper starting at 60 mg and decreased by 10 mg every week.

## 3. Discussion

MMC has been used in cancer treatment for several decades, having been approved by the FDA in 1974. MMC is an alkylating agent that produces crosslinking in DNA between mostly the guanine and cytosine pairs, which leads to disruption of DNA and RNA synthesis. Its use has been in several cancers including bladder, cervical, breast, colorectal, gastric, and pancreatic [4]. It is currently used most often in treating bladder cancer, as seen in our patient. Alkylating agents do not target specific cells, which leads to a large side-effect panel. The most common adverse reactions are gastrointestinal, with anorexia, nausea, and vomiting reported in 14% [3]. Additionally, bone marrow suppression (64%), hemolytic-uremic syndrome (< 15%), thrombotic thrombocytopenic purpura (< 15%), and fever (14%) were other adverse reactions that were seen in more than 10% of patients [3]. Our case reports an incidence of ARDS caused by drug-induced pneumonitis secondary to MMC, which postmarketing and case reports show < 1% ARDS from MMC, which makes this presentation rare and easily missed on the differential diagnosis.

There are several reported cases of pulmonary toxicity, including ARDS and hypoxic respiratory failure in patients who have received MMC (Table 1). The majority of these cases are reported in patients who received MMC in conjunction with vinca alkaloid agents. Vinca alkaloid agents bind to tubulin and inhibit microtubule formation, leading to cell arrest in the metaphase. Vinblastine, a commonly used vinca alkaloid agent, does have warnings for pulmonary toxicity. It has been reported to cause acute shortness of breath and severe bronchospasm; however, these are most frequently seen in patients who receive vinblastine with MMC [13].

In one study, a total of 25 patients of 387 were recorded to have acute dyspnea after the administration of MMC with either vinblastine (13 patients) or vindesine (12 patients) [2]. Thirteen patients had received cisplatin therapy with the MMC and vinca alkaloid. The acute dyspnea always occurred after the administration of the vinca alkaloid; only some (32%) of the patients received MMC on the day of the acute dyspnea [2]. The mean dose of MMC was 26.1 ± 7.6 *mg*/*m*^2^ at the time of symptoms. A total of 5 of the 25 patients required transfer to the ICU, with four requiring intubation and mechanical ventilation as seen in our patient. Unlike our patient, all of these patients in this study had received vinca alkaloids. The pathophysiology leading to pulmonary toxicity from the interaction between vinca alkaloid agents and MMC is not yet known.

The most similar case to ours is two patients reported to have ARDS following cytoreductive surgery and perioperative intraperitoneal chemotherapy with MMC and 5-fluorouracil for appendiceal malignancy. Both reported patients required intubation; no infectious etiology was found through testing [6]. While these two patients received 5-fluorouracil like our case, they underwent significantly different drug administrations via hyperthermic intraperitoneal chemotherapy (HIPEC). Our patient received MMC and 5-fluorouracil intravenously and is the only reported case of ARDS in this specific drug combination and type of administration. 5-Fluorouracil is a pyrimidine analog antimetabolite that interrupts DNA and RNA synthesis [14]. There is currently no reported drug interaction between 5-fluorouracil and MMC.

In a second larger study evaluating lung toxicity in MMC patients, a total of 14 patients out of 223 enrolled in MMC clinical trials were found to have MMC lung toxicity [15]. Premedication with steroids prior to MMC administration was not shown to decrease MMC lung toxicity. Of the 14 patients, five went on to have progressive MMC lung toxicity and long-term sequelae [15]. None of the patients identified in this study was reported to have ARDS like our patient.

Drug-related pulmonary toxicity remains a diagnosis of exclusion as other causes of acute hypoxic respiratory failure should be entertained including bacterial pneumonia, flash pulmonary edema, alveolar hemorrhage, and other inflammatory and autoimmune conditions which are more common etiologies of ARDS. In an immunocompromised person as described above, fungal pneumonias should also be considered.

## 4. Conclusion

MMC can rarely cause pneumonitis associated with ARDS. Documented cases have involved MMC in conjunction with vinca alkaloid agents or with 5-fluorouracil; however, the specific drug-to-drug interactions leading to ARDS are not known. This case highlights that MMC-induced pneumonitis is possible without vinca alkaloid use. This remains a diagnosis of exclusion as other causes such as bacterial pneumonia, flash pulmonary edema, alveolar hemorrhage, and other inflammatory and autoimmune conditions are more common etiologies of ARDS.

## Figures and Tables

**Table 1:** Summary of other reported cases of mitomycin-induced pulmonary toxicity.

**First author, publication year (number of cases)**	**Method of administration**	**Additional antineoplastic agents administered**	**MMC Total dose**	**Reported diagnosis**
Abel [5], 2017 (1)	Intraperitoneal	—	40 mg/m^2^	ARDS
Alonso [6], 2000 (2)	Intraperitoneal, intrapleural	5-Fluorouracil	23 mg/m^2^	ARDS
	Intraperitoneal, intrapleural	5-Fluorouracil	25 mg/m^2^	
Ballen [7], 1988 (1)	Intravenous	Vinblastine	46 mg/m^2^	ARDS
Hoelzer [8], 1986 (2)	Intravenous	Vinblastine, cisplatin	25 mg/m^2^	Acute pneumonitis
	Intravenous	Vinblastine, cisplatin	60 mg/m^2^	Acute pneumonitis
Konits [9], 1982 (2)	Intravenous	Vinblastine	—	ARDS
	Intravenous	Vinblastine	—	Acute interstitial pneumonitis
Kris [10], 1984 (3)	Intravenous	Vindesine	30 mg/m^2^	Acute dyspnea
	Intravenous	Vinblastine	50 mg/m^2^	Severe dyspnea
	Intravenous	Vindesine	30 mg/m^2^	Acute dyspnea
Luedeke [11], 1985 (1)	Intravenous	Vindesine	35 mg/m^2^	Acute hypoxic respiratory failure
Rao [12], 1985 (2)	Intravenous	Vinblastine	30 mg/m^2^	ARDS
	Intravenous	Vinblastine	68 mg/m^2^	Pulmonary edema

Abbreviation: ARDS, acute respiratory distress syndrome.

## Data Availability

Data sharing is not applicable to this article as no new data were created or analyzed in this study.
